# Prostate-derived ETS factor improves prognosis and represses proliferation and invasion in hepatocellular carcinoma

**DOI:** 10.18632/oncotarget.14924

**Published:** 2017-01-31

**Authors:** Er-Bao Chen, Shao-Lai Zhou, Xu-Guang Pang, Dan Yin, Pei-Zhen Miao, Yi Yang, Qing Chen, Kai Zhu, Dong-Mei Gao, Tian-Shu Liu, Xiao-Yi Wang, Ying-Hong Shi, Wei-Zhong Wu, Jian Zhou, Zheng-Jun Zhou, Zhi Dai

**Affiliations:** ^1^ Liver Cancer Institute, Zhongshan Hospital, Fudan University, Key Labolatory of Carcinogenesis and Cancer Invasion, Fudan University, Ministry of Education, Shanghai, China; ^2^ State Key Laboratory of Genetic Engineering, Fudan University, Shanghai, China; ^3^ Department of Thoracic Surgery, Zhongshan Hospital, Fudan University, Shanghai, China; ^4^ Department of Medical Oncology, Zhongshan Hospital, Fudan University, Shanghai, China

**Keywords:** PDEF, HCC, EMT, invasion, proliferation

## Abstract

Prostate-derived E-twenty-six (ETS) factor (PDEF), an epithelium-specific ETS transcription factor, regulates carcinogenesis and tumor progression. The prognostic importance and biologic functions in hepatocellular carcinoma (HCC) have not been established. We investigated PDEF expression in 400 HCC patients using quantitative real-time polymerase chain reaction, western blot and immunohistochemistry analysis. PDEF expression was significantly lower in tumors than in peritumoral tissues. Lower PDEF levels were associated with poorer prognosis in patients. PDEF was an independent predictor of overall survival in multivariate analysis. PDEF expression was suppressed in highly metastatic HCC cell lines, and shRNA-mediated down-regulation of PDEF in low-metastatic HCC cell lines (with high PDEF) significantly increased cellular proliferative and invasive capacity *in vitro* and *in vivo*. RNA sequencing analysis indicated that PDEF may mediate transcription of several genes involved in apoptosis and the cell cycle. PDEF modulated epithelial-mesenchymal transition by up-regulating E-cadherin expression and down-regulating Slug and Vimentin expression, thereby lowering migration and invasion abilities of HCC cells. In conclusion, PDEF is associated with proliferation and invasiveness of HCC cells. It may serve as an independent predictor of prognosis in patients with HCC.

## INTRODUCTION

Hepatocellular carcinoma (HCC) is one of the most aggressive and common malignancies [1]. It is the third most common cancer in men and fourth most common cause of malignancy-related death in China [2]. Considering the high rates of metastasis and recurrence after curative resection, understanding the mechanisms behind HCC progression and identifying new therapeutic targets are critical [3].

The human E-twenty-six (ETS) transcription factor family [4] plays important roles in cellular proliferation, apoptosis, differentiation, transformation, invasion, and oncogenesis [5–7]. Aberrant ETS gene expression has been observed in numerous malignancies, including breast, prostate, and colon cancer [8–10]. For example, ERG and ETV1 are frequently overexpressed in prostate tumorigenesis [11]. ELF5, an important member of the ETS family, promotes lung metastasis in luminal subtype breast cancer by recruiting myeloid-derived suppressor cells [12]. Emerging evidence suggests that certain ETSs are important factors implicated in tumor development and progression.

Prostate-derived ETS factor (PDEF), also called prostate-specific ETS (PSE) or SAM pointed domain-containing ETS transcription factor (SPDEF), is an ETS transcription factor originally identified and overexpressed in prostate tissue [6–8]. PDEF is down-regulated in the development of various tumors and plays a vital role in regulating carcinogenesis and tumor progression. In breast cancer, PDEF may control tumor growth and progression by regulating transcription of p21/CIP1, a cell-cycle regulatory protein [13]. PDEF may inhibit colon cancer growth and migration by targeting p21 [14]. Nevertheless, the prognostic importance of PDEF in HCC and its function in HCC carcinogenesis and metastasis remain unknown.

Herein, we evaluated PDEF expression in HCC tumor tissues using western blot and quantitative real-time polymerase chain reaction (qRT-PCR) analyses, and evaluated the prognostic significance of PDEF using tissue microarrays (TMAs) derived from HCC patients. Using specific RNA interference, we evaluated PDEF's role in HCC proliferation and invasiveness *in vitro* and *in vivo*. We also used RNA-seq to analyze potential PDEF target genes in HCC. Our data indicate that PDEF overexpression in HCC inhibits proliferation and invasion and predicts good outcomes in HCC patients.

## RESULTS

### PDEF expression is down-regulated in human HCCs

**Figure 1:**
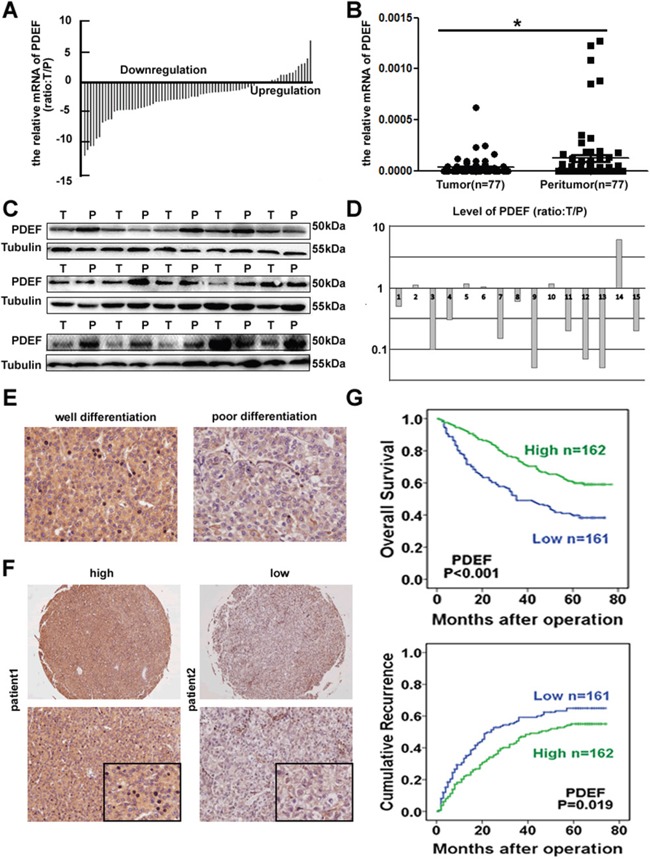
Downregulation of PDEF correlated with human HCCs **A, B.** Relative expression of PDEF among tumor and peritumoral tissues using qRT-PCR. **C, D.** The tumor tissues selected randomly exhibited low level of PDEF compared adjacent peritumoral tissues by western blot. T and P represent tumor tissue and peritumoral tissue, respectively. **E.** Representative IHC analysis of PDEF in well and poor differentiated HCC were shown. Magnification, 200X. **F.** Representative HCC tumor samples, high and low expression of PDEF. **G.** Kaplan-Meier curves of survival differences in PDEF-high and PDEF-low HCC patients in TMA. *P<0.05, **P<0.01.

PDEF mRNA expression was measured using qRT-PCR in 77 paired HCC specimens. Nearly 77.9% of tumors had reduced PDEF mRNA levels, compared with the levels in matched adjacent tissues (P<0.05; Figure 1A, 1B). Of the 77 pairs, 15 were randomly selected by random number generation (via SPSS) to undergo western blot analysis. PDEF protein was down-regulated in 10 of 15 HCC samples compared with the levels in adjacent tissues, suggesting decreased PDEF expression in tumor tissues (Figure 1C, 1D). PDEF expression was clearly reduced in poorly-differentiated tissues compared with well-differentiated tissues (Figure 1E). Thus, we observed through different methods that PDEF was down-regulated in human HCCs, suggesting a potential role for PDEF during HCC progression.

### PDEF is an independent prognosis indicator in HCC patients

We next evaluated PDEF expression using immunohistochemical staining in TMAs from 323 HCC patients. Patient clinic pathological features are listed in Table 1. In 270 out of 323 patients (83.6%) the expression of PDEF in tumor tissues was lower than that in normal adjacent tissues (Supplementary Figure 1A). The PDEF level was positively correlated with AFP (P=0.016), tumor size (P=0.030), and microvascular invasion (P=0.012) (Table 1). By final follow-up, 175 patients developed recurrent tumor and 165 died. The 3- and 5-year OS rates were 62.2% and 50.7%, respectively, and the 3- and 5-year recurrence rates were 50.2% and 59.7%, respectively. PDEF^High^ patients exhibited significantly higher1-, 3-, and 5-year survival rates than PDEF^Low^patients: 92.5% vs. 75.2%, 73.4% vs. 49.1%, 46.3%vs. 39.1%, respectively. Similarly, the median TTR and OS time were significantly longer in PDEF^High^patients than in PDEF^Low^patients: TTR, 35 mo vs.17 mo (P=0.021);OS, 63 mo vs. 35 mo (P<0.001) (Figure 1F, 1G). To identify other prognostic markers associated with OS time or TTR, we performed univariate analysis for a number of factors, as shown in Table 2. Multivariate analysis identified these independent predictors of HCC prognosis: PDEF level, tumor size, tumor encapsulation, and microvascular invasion.

**Table 1:** Correlation between the factors and clinicopathologic characteristics in HCC (n=323)

Clinicopathological indexes		PDEF ^Low^	PDEF ^High^	P
Age, years	≤50	81	85	0.698
	>50	80	77	
Sex	Female	21	25	0.539
	Male	140	137	
HBsAg	Negative	22	23	0.890
	Positive	139	139	
AFP, ng/mL	≤20	38	58	**0.016**
	>20	123	104	
γ-GT, U/L	≤54	61	70	0.330
	>54	100	92	
Liver cirrhosis	No	15	21	0.298
	Yes	146	141	
Tumor size, cm	≤5	72	92	**0.030**
	>5	89	70	
Tumor number	Single	141	135	0.279
	Multiple	20	27	
Microvascular invasion	Absence	76	99	**0.012**
	Present	85	63	
Tumor encapsulation	Complete	81	83	0.868
	None	80	79	
Tumor differentiation	I+II	120	135	0.052
	II+IV	41	27	
TNM stage	I	69	85	0.084
	II III	92	77	

HBsAg: hepatitis B surface antigen; AFP: a-fetoprotein; γ-GT: γ-glutamyl transferase; TNM: tumor-nodes metastasis.

**Table 2:** Univariate and multivariate analyses of prognostic factors with TTR and OS in HCC (n = 323)

Variables	TTR		OS	
	HR (95%CI)	P	HR (95%CI)	P
**Univariate analysis**				
Age, years (≤50 vs. >50)	1.023 (0.760-1.377)	0.879	0.828 (0.610–1.125)	0.228
Sex (female vs. male)	1.863 (1.143–3.036)	**0.013**	1.757 (1.049–2.942)	**0.032**
HBsAg (negative vs. positive)	1.007 (0.659–1.538)	0.974	1.039 (0.669–1.614)	0.866
AFP, ng/mL (≤20 vs. >20)	1.155 (0.835–1.597)	0.385	1.548 (1.083–2.211)	**0.016**
γ-GT, U/L (≤54 vs. >54)	1.329 (0.978–1.805)	0.069	1.737 (1.253–2.409)	**0.001**
Liver cirrhosis (no vs. yes)	1.102 (0.677–1.795)	0.696	1.308 (0.769–2.224)	0.332
Tumor size, cm (≤5 vs. >5)	1.817 (1.346–2.452)	**0.000**	2.482 (1.806–3.412)	**0.000**
Tumor number (single vs. multiple)	1.362 (0.907–2.044)	0.136	1.517 (1.025–2.243)	**0.037**
Microvascular invasion (no vs. yes)	1.915 (1.420–2.583)	**0.000**	2.479 (1.815–3.388)	**0.000**
Tumor encapsulation (complete vs. none)	1.679 (1.246–2.263)	**0.001**	1.715 (1.260–2.336)	**0.001**
Tumor differentiation (I+II vs. II+IV)	1.238 (0.870–1.763)	0.236	1.581 (1.119–2.233)	**0.009**
TNM stage (I vs. II III)	2.072 (1.527–2.814)	**0.000**	2.683 (1.935–3.720)	**0.000**
PDEF (low vs. high)	0.705 (0.523–0.949)	**0.021**	0.510 (0.374–0.697)	**0.000**
Multivariate analysis				
Sex (female vs. male)	1.692 (1.029-2.781)	**0.038**	1.426 (0.848-2.404)	0.183
AFP, ng/mL (≤20 vs. >20)	1.039 (0.740-1.459)	0.826	1.143 (0.784-1.665)	0.488
γ-GT, U/L (≤54 vs. >54)	1.158(0.838-1.599)	0.373	1.427 (1.009-2.018)	**0.044**
Tumor size, cm (≤5 vs. >5)	1.531(1.112-2.108)	**0.009**	1.933 (1.381-2.705)	**0.000**
Tumor number (single vs. multiple)	1.252(0.824-1.901)	0.290	1.341 (0.896-2.006)	0.153
Microvascular invasion (no vs. yes)	1.537(1.113-2.122)	**0.009**	1.926 (1.383-2.683)	**0.000**
Tumor encapsulation (complete vs. none)	1.578(1.158-2.148)	**0.004**	1.578 (1.143-2.180)	**0.006**
Tumor differentiation (I+II vs. II+IV)	1.192(0.823-1,727)	0.351	1.482 (1.031-2.129)	**0.033**
PDEF (low vs. high)	0.776(0.468-0.860)	0.101	0.584 (0.424-0.804)	**0.001**

TTR: time to recurrence; OS: overall survival; HBsAg: hepatitis B surface antigen; AFP: a-fetoprotein; γ-GT: γ-glutamyl transferase; TNM: tumor-nodes metastasis; HR: hazard ratio; CI: confidential interval.

### PDEF regulates cancer cell proliferation by regulating apoptosis and the cell cycle *in vitro*

**Figure 2:**
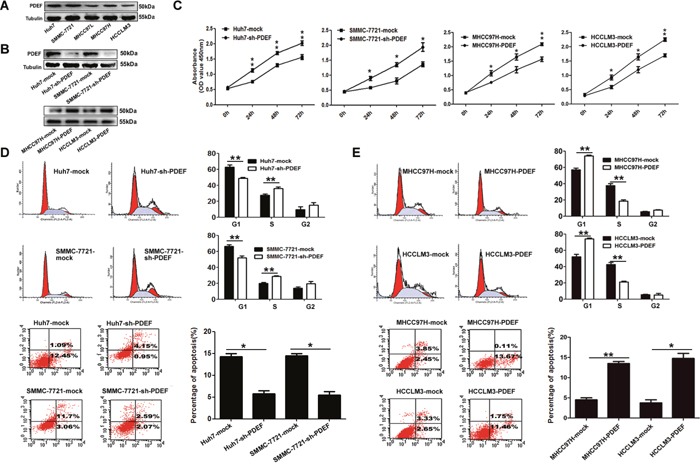
PDEF regulates cellular proliferation and anti-apoptosis of HCC cell lines **A.** PDEF protein is downregulated in HCC cell lines. Western blot was used to quantify the endogenous levers levels of PDEF protein. Tubulin served as the control. **B.** PDEF expression was confirmed using western blot analysis of stably transfected and of parent cells. **C.** Cell proliferation tested test by using cell counting kit 8 after knockdown or overexpression of PDEF in HCC cells. Quantitation of was done by reporting the observed OD values for tumor cell OD value. **D.** Huh7 and SMMC-7721 were transfected with PDEF-shRNA or mock for 48 hours then analyzed by flow cytometry. **E.** MHCC97H and HCCLM3 were transfected with PDEF forced expression or mock for 48 hours then analyzed by flow cytometry. Percentages (%) of cell subpopulations at different stages of cell cycles and apoptosis are list within the figures. Data was reported as the mean+ values with standard deviations from least three independent experiments, with each assay was performed in triplicate times. *P<0.05, **P<0.01.

PDEF expression was significantly reduced in highly metastatic cell lines (MHCC97H and HCCLM3) compared with low-metastatic HCC cell lines (SMMC-7721 and Huh7) (Figure 2A). Huh7 and SMMC-7721 cells (with high PDEF levels) were transfected with short hairpin RNA (shRNA) directed against PDEF, and stable knockdown of PDEF expression was confirmed by western blot testing (Figure 2B). *In vitro* cell proliferation assay showed that PDEF down-regulation in Huh7 and SMMC-7721 cells caused significantly increased cell proliferation (Figure 2C). During cell cycle analysis, Huh7 with PDEF shRNA treated (Huh7-shRNA-PDEF) lines and SMMC-7721-shRNA-PDEF exhibited higher numbers of S-phase cells than control lines. The proportion of apoptosis cells was significantly reduced in Huh7-shRNA-PDEF and SMMC-7721-shRNA-PDEF compared with the control (Figure 2D). To confirm these results, we forced expression of PDEF in highly malignant HCC (MHCC97H and HCCLM3) (Figure 2B). As result, we found that cell proliferation was inhibited, the percentages of S-phase decreased and the apoptosis ratio increased in PDEF-transfected cells, compared with control lines (Figure 2C, 2E).

### PDEF knockdown promotes cellular mobility and invasion *in vitro*

**Figure 3:**
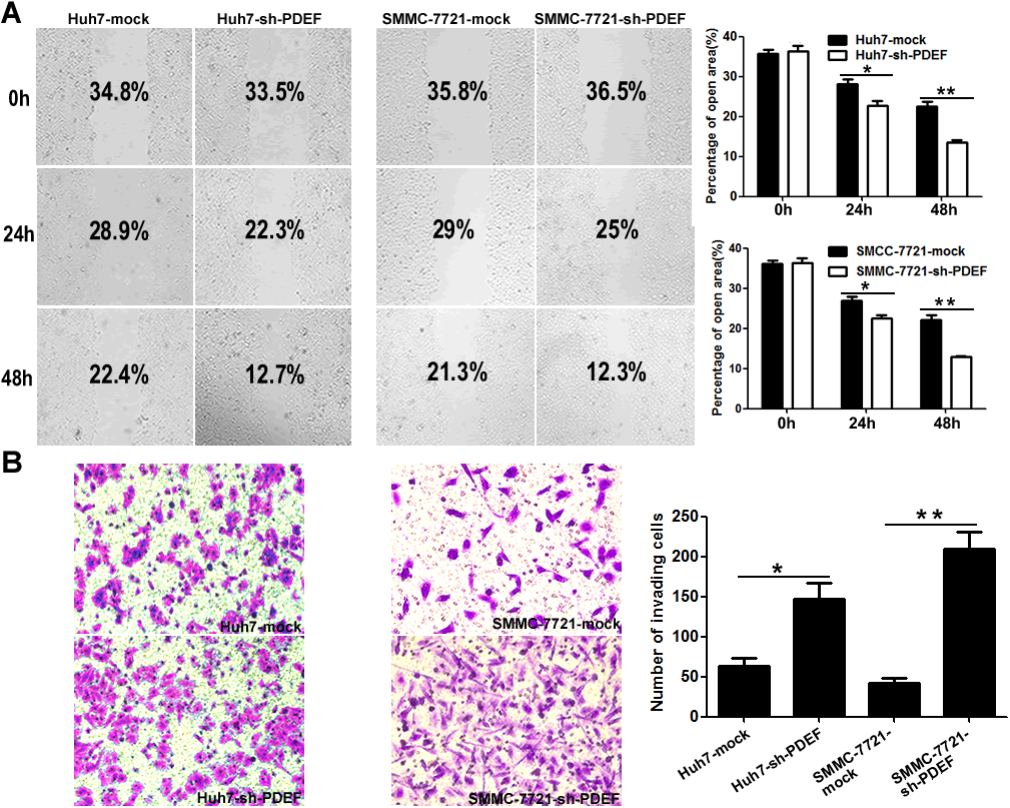
PDEF deficiency enhances cellular mobility and invasion *in vitro* **A.** Wound healing migration assays indicated the influence of PDEF loss on cellular mobility. Quantification of the percentages (%) of empty area were quantified and were shown as a bar graph. Magnification, 200X. **B.** Transwell invasion assay, and quantification of cell invasion was is displayed. Magnification, 200X. The results are mean+ SD of at least three independent experiments. *P<0.05, **P<0.01.

In wound-healing assays, post-wound microscopic examination revealed a significantly higher wound with Huh7-shRNA-PDEF cells than Huh7 mock cells (P<0.05; Figure 3A). In transwell invasion assays, the number of invasive cells was significantly higher in shRNA-PDEF-Huh7 than in the control (146.7±20.3vs.63.3±10.1, P<0.05; Figure 3B). SMMC-7721 cells exhibited similar results. Collectively, *in vitro* assay data showed that PDEF knockdown promotes cell proliferation and invasion.

### PDEF down-regulation promotes tumor growth and metastasis *in vivo*

**Figure 4:**
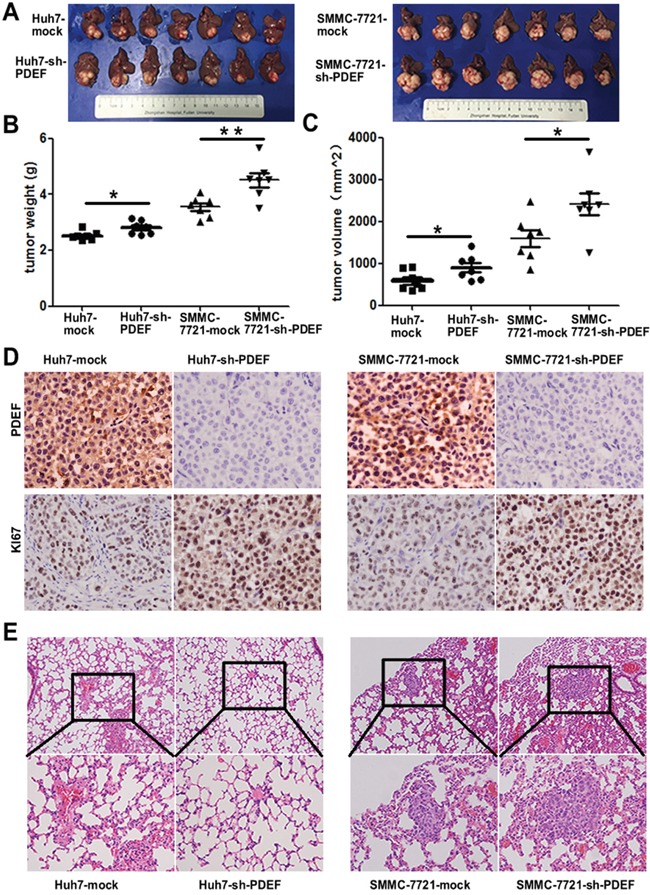
Knockdown of PDEF enhances tumor growth and metastasis *in vivo* **A.** The pictures of tumors are involved in the study. **B, C.** The weight and volume of tumors was measured after surgery performed. **D.** Representative images of tumor samples from xenograft nude mouse models stained with Ki67. **E.** The representative images of H&E stain results of lung tissue from each group. *P<0.05, **P<0.01. Magnification, 200X.

When shRNA-PDEF-SMMC-7721 was injected into nude mice, mean tumor weight of shRNA-PDEF-SMMC-7721-derived xenografts was 4.50±0.25g, which was significantly greater than the weight of SMMC-7721-mock derived xenografts(3.55±0.13g). Similarly, mean tumor weight of shRNA-PDEF-Huh7-derived xenografts was greater than that of Huh7-mock-derived tumors (2.80±0.09 g vs. 2.52±0.06g) (Figure 4A, 4B). Tumor volume data showed similar results (Figure 4C). As we have known, Ki67 was one of the most important marker of cell proliferation [15]. In our study, we found the increased expression of Ki67 in the shRNA-PDEF group compared with the mock group (Figure 4D). In addition, pulmonary metastases occurred in 4 of 7 of SMMC-7721-shRNA-PDEF-SMMC-7721 mice and only 1 of 7 SMMC-7721-mock mice. No pulmonary metastasis occurred in shRNA-PDEF-Huh7 or Huh7-mock mice (Figure 4E). These *in vivo* results indicate that PDEF deficiency dramatically increases tumor growth and metastasis.

### PDEF potential regulation genes are identified in HCC cell lines

**Figure 5:**
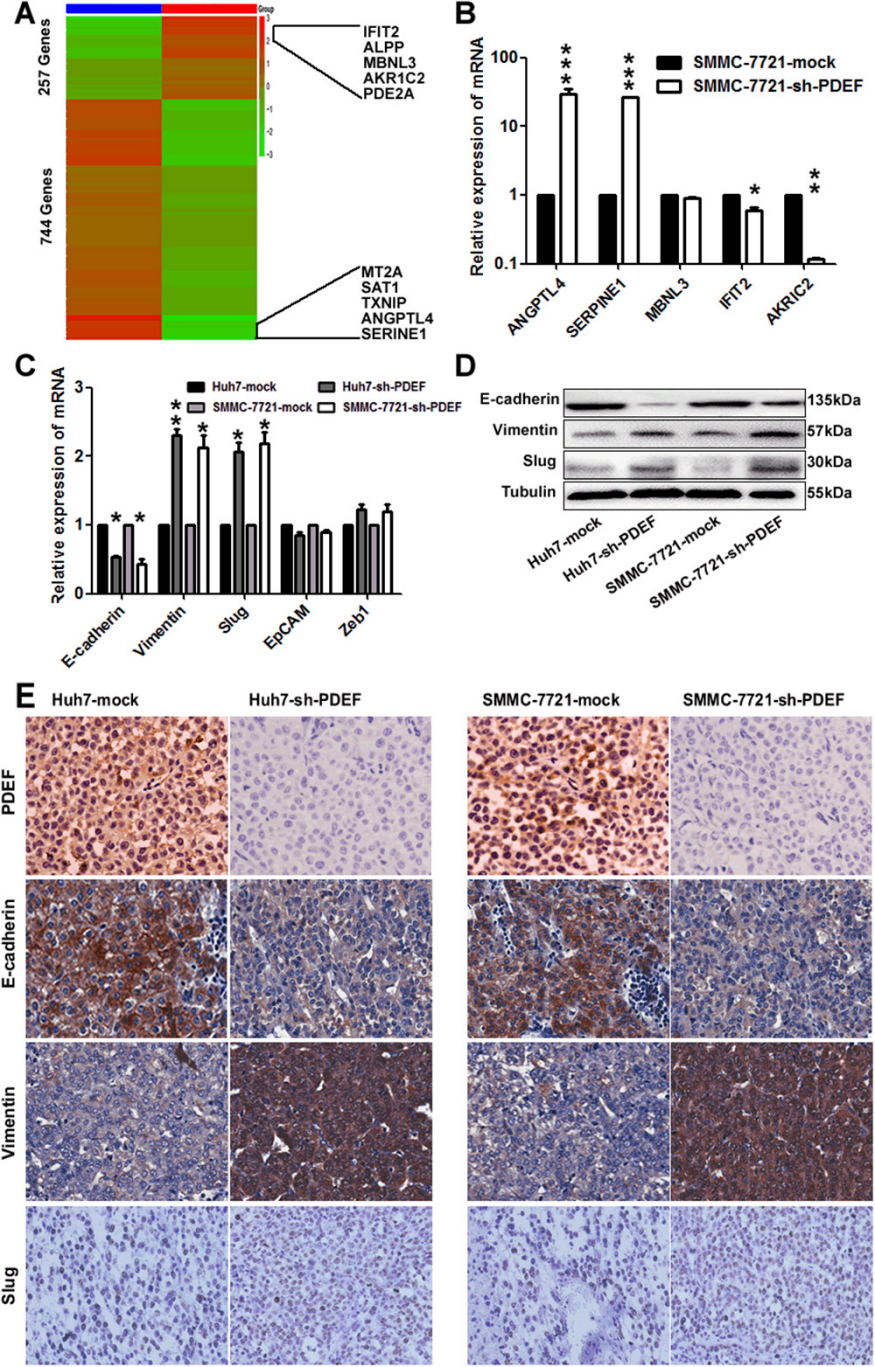
The latent modulated genes of PDEF was represented in HCC by RNA-sequencing **A.** A heat map represents hierarchical clustering of 1001 significant differentially expressed mRNA. The scale bar at the right depicts the standard deviation from the mean. The top five genes and the last five genes were labelled within the panel. **B.** The random selected differentially expressed genes in the RNA-sequencing was verified in SMMC-7721 cell by qRT-PCR. The data are shown as the means+ SD obtained in three independent experiments. **C, D.** The expression of PDEF target gene detected by qRT-PCR and western blot. **E.** Representative images from xenografts tumor samples stained with PDEF, E-cadherin, Vimentin and Slug. *P<0.05, **P<0.01, ***P<0.001. Magnification, 200X.

To explore the mechanism whereby PDEF down-expression promotes HCC proliferation and metastasis, we performed transcriptome sequencing analysis of mRNA levels in PDEF-shRNA-SMMC-7721 cells and SMMC-7721-mock cells. Two distinct mRNA expression clusters were detected by hierarchical clustering analysis (Figure 5A). Bioinformatic analyses revealed 257 down-regulated genes and 744 up-regulated genes in PDEF-shRNA-SMMC-7721 cells, compared with control cells. KEGG analysis showed that genes regulated by PDEF were involved in the tumor necrosis factor signaling pathway and apoptosis. There were 140 targets with an average of ≥10 transcripts per million of 1001 targets. We randomly selected five genes to validate the RNA-seq results from the five top-ranked and lowest-ranked genes by real-time PCR (Figure 5A). mRNA levels of IFIT2 and AKR1C2, which participate in cell apoptosis, were significantly decreased in PDEF-shRNA-SMMC-7721. SERPINE1 [16] and ANGPTL4 [17], which are involved in activating the PI3AKT signaling pathway and promoting angiogenesis, were prominently increased in PDEF-silenced cells. MBNL3 [18], which interacts with CD44, declined only slightly in PDEF-knockdown cells (Figure 5B).

Accumulating evidence from experimental and clinical studies suggests that epithelial-mesenchymal transition (EMT) serves an important function in tumor invasion and metastasis. Recent studies have also reported that PDEF may modulate the expression of EMT-related genes [19, 20]. qRT-PCR experiments showed that cellular levels of E-cadherin were significantly decreased and Vimentin and Slug were significantly increased after PDEF knockdown. Levels of other modulators were not significantly altered (Figure 5C). These results were similar to those achieved using RNA-seq. The factors with changed profiles were confirmed by western blot analysis (Figure 5D). And then we examined the expression of these genes in the shRNA-derived xenografts. As a result, we found that the expression of Vimentin and Slug were increased in the shRNA-derived xenografts when compared with the control group and the expression of E-cadherin was decreased (Figure 5E). Together, these data suggest that PDEF regulates target genes involved in HCC progression via mediating transcription.

## DISCUSSION

PDEF plays important roles in normal biological processes and cancer development. Emerging evidence indicates that PDEF dysregulation is involved in tumorigenesis and progression of tumors, including prostate [21], breast [22], and colon cancers [23]. The prognostic significance of PDEF and roles of PDEF in HCC progression have been heretofore unreported. This study demonstrated a correlation between PDEF expression and prognosis of HCC patients. Immunohistochemistry analyses examining PDEF protein levels in TMAs showed that low PDEF expression was associated with lower OS rates in HCC patients. Multivariate analysis confirmed PDEF as an independent predictor of OS time. These results are consistent with previous findings in prostate [21] and breast cancer [22], and they strongly indicate that PDEF may act as both a biomarker of tumor invasion and predictor of prognosis in HCC.

Previous studies have reported contradictory results, with PDEF functioning as a tumor suppressor or as an oncogene, depending on the tissue and cell type. This is the first report providing laboratory evidence that PDEF has tumor suppressive activity in human HCC. We used an extensive collection of HCC tumors to demonstrate that PDEF expression was lower in HCC than peritumoral tissues at the mRNA, as well as protein, level and PDEF expression was reduced in poorly-differentiated tissues compared with well-differentiated tissues. Moreover, PDEF was expressed preferentially in low-metastatic HCC cell lines. When shRNA specific for PDEF mRNA were transfected into low-metastatic HCC cells, the resultant down-regulated PDEF expression promoted cell proliferation and invasiveness. In our orthotopic xenograft model, PDEF knockdown in HCC cells markedly increased tumor size and rate of lung metastases. Thus, PDEF may serve vital role in regulating HCC growth and metastasis.

Interestingly, there are various expression patterns of PDEF depending on the different cancer types. In breast cancer, PDEF expression is decreased at protein level, but not mRNA level, when microRNA was used to target PDEF [24]; However, in colon cancer, it is decreased in tumor samples at both protein and mRNA level [14]. The reason how the difference is made is difficult to be explained. Upon our results, the expression of PDEF in HCC is consistent with that in colon cancer in a way. PDEF has been implicated in many biological functions crucial for cancer development, including proliferation [25], apoptosis, invasion [26], metastases [20, 27], angiogenesis, and differentiation [28]. We found that down-regulated PDEF expression induced cell proliferation *in vitro* by increasing the number of S-phase cells and induced tumor growth *in vivo*, consistent with previous results for other tumors [29, 30]. When it was overexpressed in highly-malignant HCC cell lines with low level of PDEF, inhibition of cell proliferation and induction of apoptosis were observed. In breast cancer, PDEF may control tumor growth and progression by regulating transcription of p21/CIP1, a cell-cycle regulatory protein [13]. To understand the function of PDEF in HCC progression, we conducted RNA sequencing to explore its target genes. We identified several cell cycle-related genes regulated by PDEF, including CDKN2D and CDKN1C. These genes have been mainly reported as regulators of the G1 to S-phase transition, strongly suggesting that PDEF regulates the cell cycle of HCC cells by transcriptional regulation.

We also discovered that PDEF regulated IFIT2, AKR1C2, SERPINE1, ANGPTL4, and MBNL3 genes. GO analysis revealed that these genes in the top rank of change fold were involved in cancer-related pathways. These results were consistent with the mechanism of PDEF underlying breast cancer [19]. The function of these genes in cancer progression has been documented in several studies. For example, AKR1C2 may facilitate cancer cell autophagy and apoptosis, and IFIT2 inhibits cell motility and invasiveness by EMT [31–33]. SERPINE1 inhibits differentiation, and ANGPTL4 regulates EMT via the ERK signaling pathway. MBNL3 enhances cell reprogramming to self-renewal [18, 34, 35]. Our results revealed that PDEF knockdown in Huh7 and SMMC-7721 cells contributes to cell invasiveness *in vitro* and lung metastasis *in vivo*, combined with decreased E-cadherin and increased Vimentin and Slug. Thus, HCC cells with low-level PDEF likely become more invasive by undergoing EMT. In our study, to demonstrate the target genes that PDEF regulated, we performed RNA-seq in both SMMC-7721-shRNA-PDEF cell line and mock cell line. We selected ten most obviously changed genes, and picked up five of them for further verification. We found that ANGPTL4, SERPINE1, IFIT2, AKRIC2 obviously changed with statistical significance between SMMC-7721-shRNA-PDEF cell line and mock cell line. But we can't distinguish which genes are directly regulated by PDEF or indirectly without chromatin immunoprecipitation assay-PCR (CHIP-PCR). In our future work, we plan to choose those genes with greater potential suggested by bioinformatics analysis, and perform ChIP on them.

In conclusion, our study demonstrates that lower PDEF expression in HCC tissue correlates with poorer prognosis and may promote proliferation, anti-apoptosis, and invasion of HCC cells through regulating various genes at the transcriptional level. Collectively, our results indicate that PDEF likely produces tumor suppression in HCC.

## MATERIALS AND METHODS

### Cell lines, cell culture, and animals

We used five human HCC cell lines. Three had high metastatic potential (MHCC97L, MHCCL97H, and HCCLM3); they were established at our institute [36]. Two with low metastatic capabilities (Huh7 and SMMC-7721) were purchased from the Institute of Biochemistry and Cell Biology, Chinese Academy of Sciences, Shanghai, China. All cell lines were cultured with high-glucose Dulbecco's modified eagle medium (DMEM) supplemented with 10% fetal bovine serum (FBS), 100U/mL penicillin, and 0.1μg/mL streptomycin and incubated at 37°C with 5% carbon dioxide. All tissue culture reagents were obtained from Sigma (USA). Cell morphology was monitored closely to ensure no abnormalities developed.

CMV-MCS-EGFP-SV40-Neomycin-RNAi-PDEF and CMV-MCS-EGFP-SV40-Neomycin-RNAi-mock plasmids were purchased from Shanghai Genechem Company (Shanghai, China). CMV-MCS-EGFP-SV40-Neomycin-RNAi-PDEF plasmid was transfected into Huh7 and SMMC-7721 according to the lentiviral instructions. CMV-MCS-EGFP-SV40-Neomycin-RNAi-mock plasmids were used as controls. ShRNA-PDEF and mock cells were incubated with 10 μg/mL puromycin for three generations. Stably transfected clones were confirmed by western blotting for PDEF.

We used 4- to 6-week-old male BALB/C nu/nu mice (Shanghai Institute of Material Medicine, Chinese Academy of Science). They were maintained under pathogen-free conditions and received humane care. All animal protocols complied with the “Guide for the Care and Use of Laboratory Animals” established by the National Academy of Sciences and issued by the National Institutes of Health (NIH publication 86-23, 1985revision).

### Patients and follow-up

Two cohorts, derived from 400 patients with HCC, were involved in this study. Cohort 1 included 77 consecutive patients undergoing curative resection in January-June 2007 at our institute (Zhongshan Hospital, Fudan University). Their tumor and adjacent normal tissues were used for qRT-PCR analysis. Cohort 2 included 323 randomly selected patients who underwent curative resection at our institute in 2003-2004 [37]. Their tumor and adjacent tissues were used for TMA analysis. The histopathological diagnosis was based on World Health Organization criteria: typical appearance on magnetic resonance imaging or computed tomography, plus elevated alpha-fetoprotein (AFP). Ethics committee-approved forms consenting to the use of any extra tissue for research were obtained from all patients. Tumor differentiation histological grade was graded according to the Edmondson and Steiner classification system. Liver function was categorized using the Child-Pugh system. Tumor stage was determined using the 2010 International Union Against Cancer tumor-node-metastasis system.

Cohort 1 patients were followed postoperatively until March 15, 2009, and cohort 2 patients were followed until March 21, 2011. The median follow-up period of all patients was 48 months (1.0-52.5 months). Overall survival (OS) time was defined as the period from the surgery date until the date of death. Time to recurrence (TTR) was the period from the surgery date until the date of first reported recurrence (intrahepatic or distant). Patients were excluded if they died of a non-HCC-related cause before recurrence was detected. For patients without recurrence by the time of last follow-up or death, TTR was determined using the final follow-up or death date.

### Tissue microassay and immunohistochemistry

Pairs of 323 specimens diagnosed as HCC plus normal tissue adjacent to the tumors were formalin-fixed, paraffin-embedded, then stored at −20°C. For analysis, the tissue sections were dewaxed, followed by rehydration using dimethylbenzene and hydrous ethanol. Endogenous peroxidase activity was blocked by incubation with 3% hydrogen peroxide for 15 min. Sections submerged in 10 mmol/L citrate at pH 6 were microwaved on high power for 7 min, followed by washing with phosphate-buffered saline (PBS) for 3 min. The sections were subsequently incubated overnight at 4°C with PDEF antibody (Catalog#ab197375, Rabbit polyclonal PDEF antibody, Abcam, USA) diluted 1:200 with blocking solution. This was followed by three PBS washes, after which the slides were exposed to horse anti-rabbit antibody at room temperature for 1 h.

Immunohistochemistry staining was conducted using the avidin-biotin complex method. A position stain was developed using a-diaminobezidine. Data were collected regarding signal intensity and percentage of staining. When PDEF staining was unclear, the cells were categorized as high or low by comparing them with cells on the corresponding benign (adjacent) tissue slides. Negative controls were analyzed in a similar manner, except for the use of primary antibodies.

### RNA isolation, qRT-PCR, and western blot analysis

Total RNA extraction and isolation were performed using Trizol Reagent (Invitrogen, USA) and standard procedures described in the Supplementary. Reverse transcription of 2 μg total RNA was performed using PrimeScript PT Reagent Kit (TaKaRa, Japan). mRNA expression levels were measured by qRT-PCR utilizing SYBR Premix Ex Taq II (TaKaRa, Japan). PCR amplification involved an initial 2-min step at 95°C, then 40 cycles of the following: 15 sec at 95°C, 30 sec at 60°C, and 30 sec at 70°C. Glyceraldehyde-3-phosphate dehydrogenase (GAPDH) was employed as an endogenous control to normalize the quantity of RNA between samples. The primers used for PCR amplification were 5′-ATGGGCAGCGCCAGCCCGGGTC-3′ (forward) and 5′-TCAGATGGGGTGCACGAACTGGT-3′ (reverse) for PDEF and 5′-AGCCACATCGCTCAGACA-3′ (forward) and 5′-GCCCAATACGACCAAATCC-3′ (reverse) for GAPDH.

Cells at 90% confluence were washed three times with ice-cold PBS, then extracted in PIPA buffer containing protease inhibitors. Total protein extraction and standard procedures were described in the Supplementary. All samples were added to 5X loading buffer and boiled for 5 min. Protein (50 μg) extracted from each sample was resolved by 10% sodium dodecyl sulfate-polyacrylamide gel electrophoresis (SDS-PAGE). These extracted samples were transferred to a polyvinylidene difluoride membrane (Millipore, USA), which was blocked by incubation at room temperature for 1 h with TBST buffer (20 mM Tris/HCl, pH 7.5; 0.137M NaCI; 0.05% Tween-20) containing 5% nonfat skim milk. The membrane was then probed with polyclonal PDEF antibody (Abcam, ab197375, USA) diluted 1:200 with TBST buffer, followed by addition of a secondary antibody. Protein was detected using enhanced chemiluminescence. Tubulin (Cell Signaling Technology, USA) was used as a loading control. Each experiment was conducted three times.

### Cell proliferation assay, cell migration, and cell invasion assay

Using a 96-well culture plate, 1000 cells were plated onto each well and incubated for 0, 24, 48, and 72 h at 37°C under 5% carbon dioxide. At each time, 10μL of cell counting kit-8 solution (Dojindo, Japan) were added, and absorbance at 450nm was determined using an Infinite 200 spectrometer. We performed each assay in triplicate and conducted three independent trials.

Cell migration was examined by the scratch wound assay. After culturing for 2 days to establish a tight monolayer, the cells were deprived of serum for 16 h. The monolayer was then wounded using a 10-μL plastic pipette tip. The cells were washed twice to remove cell debris, followed by incubation with standard culture medium (containing serum) at 37°C. Cells migrating to the wound front were photographed after 24 and 48h using an inverted microscope (Leica, Hesse, Germany). Migration capacity was quantified by measuring the percent open area.

After suspension in 150 μL DMEM medium with 1% FBS, treated and untreated (control) HCC cells were seeded in triplicate onto the upper chamber of a transwell insert, with 50,000 cells per well. Medium containing 10% FBS was placed in the lower chamber to function as a chemoattractant. After 48 h incubation, upper chamber cells were extracted by scraping, and those cells left on the lower insert surface underwent fixation with 4% paraformaldehyde, followed by 10 min of crystal violet staining. The numbers of cells in 10 random microscope fields (magnification, 200X) were counted. Error bars in figure represent the standard deviation of three distinct data sets.

### Flow cytometry for apoptosis and cell cycle analysis

Cells were harvested after 48 h, then fixed and dehydrated for 24 h using 70% ethanol at −20°C. After washing twice with PBS, the cells were resuspended in 500 μL solution containing 0.5 mg/mL propidium iodide and 1mg/mL RNase A (Sigma-Aldrich, USA). This solution was kept in the dark before analysis. After 48 h, the cells were again harvested and washed twice with PBS. They were subsequently centrifuged, then stained simultaneously with Alexa Flur488 Annexin V and PI (Life Technology, USA). Stained cells underwent flow cytometry.

### *In vivo* assays for tumor growth and metastasis

The transfected and parent HCC cells were suspended in 100 uL DMEM (serum-free) and implanted subcutaneously into the flanks of nude mice. When the tumors were approximately 1 cm long (about 4 w post-injection), they were excised and then cut into small, standard-size pieces (2×2×2 mm^3^) for transplantation into nude mice livers (6 in each group). The mice were evaluated every 5 days and euthanized after 5 w. Tumor tissue sections were prepared, and immunoreactivity was analyzed. The lungs were excised, then embedded in paraffin. The number of lung metastases was counted while viewed under a microscope.

### RNA sequencing

RNA was sequenced by following the manufacturer's directions. Total RNA was extracted from approximately10^6^cells (shRNA-PDEF-SMMC-7721 and SMMC-7721-mock) using Trizol reagent (Invitrogen), then analyzed with a Nanodrop spectrophotometer (Thermo, USA) to determine the quantity and concentration of RNA following gel electrophoresis. A TruSeq PE Cluster Kit (Illumina, USA) was used for 3′-adaptor ligation, 5′-adaptor ligation, reverse transcription, and PCR amplification, and samples were PAGE-purified to construct a cDNA library. cDNA library construction and Solexa sequencing were performed using Hiseq2500 (Illumina) at Genergy Biotechnology Limited (Shanghai, China).

### Statistical analysis

Two-sided paired Student's t-tests were used to compare quantitative samples data. OS and tumor-free survival were estimated using the Kaplan-Meier method. The log-rank test was employed to assess survival differences between patients with PDEF-positive and PDEF-negative cancers. Chi-square or Fisher exact tests were used for categoricaldata. Univariate and multivariate analyses were based on Cox proportional hazard regression models. Data are presented as mean±standard deviation. P≤0.05 was considered statistically significant. SPSS software for Windows (version 21, USA) was used for all analyses.

## SUPPLEMENTARY MATERIALS FIGURE



## References

[1] Chaffer CL, Weinberg RA (2011). A perspective on cancer cell metastasis. Science.

[2] Chen W, Zheng R, Baade PD, Zhang S, Zeng H, Bray F, Jemal A, Yu XQ, He J (2016). Cancer statistics in China, 2015. CA Cancer J Clin.

[3] Hanahan D, Weinberg RA (2011). Hallmarks of cancer: the next generation. Cell.

[4] Cho JY, Lee M, Ahn JM, Park ES, Cho JH, Lee SJ, Kim BG, Heo SH, Park HJ, Zerbini LF, Hwang D, Libermann TA (2009). Proteomic analysis of a PDEF Ets transcription factor-interacting protein complex. J Proteome Res.

[5] Feldman RJ, Sementchenko VI, Watson DK (2003). The epithelial-specific Ets factors occupy a unique position in defining epithelial proliferation, differentiation and carcinogenesis. Anticancer Res.

[6] Oettgen P, Finger E, Sun Z, Akbarali Y, Thamrongsak U, Boltax J, Grall F, Dube A, Weiss A, Brown L, Quinn G, Kas K, Endress G (2000). PDEF, a novel prostate epithelium-specific ets transcription factor, interacts with the androgen receptor and activates prostate-specific antigen gene expression. J Biol Chem.

[7] Feldman RJ, Sementchenko VI, Gayed M, Fraig MM, Watson DK (2003). Pdef expression in human breast cancer is correlated with invasive potential and altered gene expression. Cancer Res.

[8] Thompson HG, Harris JW, Wold BJ, Lin F, Brody JP (2003). p62 overexpression in breast tumors and regulation by prostate-derived Ets factor in breast cancer cells. Oncogene.

[9] Galang CK, Muller WJ, Foos G, Oshima RG, Hauser CA (2004). Changes in the expression of many Ets family transcription factors and of potential target genes in normal mammary tissue and tumors. J Biol Chem.

[10] Thelen P, Jarry H, Ringert RH, Wuttke W (2004). Silibinin down-regulates prostate epithelium-derived Ets transcription factor in LNCaP prostate cancer cells. Planta Med.

[11] Lunardi A, Varmeh S, Chen M, Taulli R, Guarnerio J, Ala U, Seitzer N, Ishikawa T, Carver BS, Hobbs RM, Quarantotti V, Ng C, Berger AH (2015). Suppression of CHK1 by ETS family members promotes DNA damage response bypass and tumorigenesis. Cancer Discov.

[12] Gallego-Ortega D, Ledger A, Roden DL, Law AM, Magenau A, Kikhtyak Z, Cho C, Allerdice SL, Lee HJ, Valdes-Mora F, Herrmann D, Salomon R, Young AI (2015). ELF5 drives lung metastasis in luminal breast cancer through recruitment of Gr1+ CD11b+ myeloid-derived suppressor cells. PLoS Biol.

[13] Schaefer JS, Sabherwal Y, Shi HY, Sriraman V, Richards J, Minella A, Turner DP, Watson DK, Zhang M (2010). Transcriptional regulation of p21/CIP1 cell cycle inhibitor by PDEF controls cell proliferation and mammary tumor progression. J Biol Chem.

[14] Moussa O, Turner DP, Feldman RJ, Sementchenko VI, McCarragher BD, Desouki MM, Fraig M, Watson DK (2009). PDEF is a negative regulator of colon cancer cell growth and migration. J Cell Biochem.

[15] Scholzen T, Gerdes J (2000). The Ki-67 protein: from the known and the unknown. J Cell Physiol.

[16] Pavón MA, Arroyo-Solera I, Téllez-Gabriel M, León X, Virós D, López M, Gallardo A, Céspedes MV, Casanova I, López-Pousa A, Mangues MA, Quer M, Barnadas A, Mangues R (2015). Enhanced cell migration and apoptosis resistance may underlie the association between high SERPINE1 expression and poor outcome in head and neck carcinoma patients. Oncotarget.

[17] Hu K, Babapoor-Farrokhran S, Rodrigues M, Deshpande M, Puchner B, Kashiwabuchi F, Hassan SJ, Asnaghi L, Handa JT, Merbs S, Eberhart CG, Semenza GL, Montaner S, Sodhi A (2016). Hypoxia-inducible factor 1 upregulation of both VEGF and ANGPTL4 is required to promote the angiogenic phenotype in uveal melanoma. Oncotarget.

[18] Holm F, Hellqvist E, Mason CN, Ali SA, Delos-Santos N, Barrett CL, Chun HJ, Minden MD, Moore RA, Marra MA, Runza V, Frazer KA, Sadarangani A, Jamieson CH (2015). Reversion to an embryonic alternative splicing program enhances leukemia stem cell self-renewal. Proc Natl Acad Sci U S A.

[19] Findlay VJ, Turner DP, Yordy JS, McCarragher B, Shriver MR, Szalai G, Watson PM, Larue AC, Moussa O, Watson DK (2011). Prostate-derived ETS factor regulates epithelial-to-mesenchymal transition through both SLUG-dependent and independent mechanisms. Genes Cancer.

[20] Steffan JJ, Koul HK (2011). Prostate derived ETS factor (PDEF): a putative tumor metastasis suppressor. Cancer Lett.

[21] Ghadersohi A, Sharma S, Zhang S, Azrak RG, Wilding GE, Manjili MH, Li F (2011). Prostate-derived Ets transcription factor (PDEF) is a potential prognostic marker in patients with prostate cancer. Prostate.

[22] Buchwalter G, Hickey MM, Cromer A, Selfors LM, Gunawardane RN, Frishman J, Jeselsohn R, Lim E, Chi D, Fu X, Schiff R, Brown M, Brugge JS (2013). PDEF promotes luminal differentiation and acts as a survival factor for ER-positive breast cancer cells. Cancer Cell.

[23] Deves C, Renck D, Garicochea B, da Silva VD, Giulianni Lopes T, Fillman H, Fillman L, Lunardini S, Basso LA, Santos DS, Batista EL (2011). Analysis of select members of the E26 (ETS) transcription factors family in colorectal cancer. Virchows Arch.

[24] Findlay VJ, Turner DP, Moussa O, Watson DK (2008). MicroRNA-mediated inhibition of prostate-derived Ets factor messenger RNA translation affects prostate-derived Ets factor regulatory networks in human breast cancer. Cancer Res.

[25] Sood AK, Wang J, Mhawech-Fauceglia P, Jana B, Liang P, Geradts J (2009). Sam-pointed domain containing Ets transcription factor in luminal breast cancer pathogenesis. Cancer Epidemiol Biomarkers Prev.

[26] Turner DP, Findlay VJ, Kirven AD, Moussa O, Watson DK (2008). Global gene expression analysis identifies PDEF transcriptional networks regulating cell migration during cancer progression. Mol Biol Cell.

[27] Turcotte S, Forget MA, Beauseigle D, Nassif E, Lapointe R (2007). Prostate-derived Ets transcription factor overexpression is associated with nodal metastasis and hormone receptor positivity in invasive breast cancer. Neoplasia.

[28] Noah TK, Kazanjian A, Whitsett J, Shroyer NF (2010). SAM pointed domain ETS factor (SPDEF) regulates terminal differentiation and maturation of intestinal goblet cells. Exp Cell Res.

[29] Turner DP, Findlay VJ, Moussa O, Semenchenko VI, Watson PM, LaRue AC, Desouki MM, Fraig M, Watson DK (2011). Mechanisms and functional consequences of PDEF protein expression loss during prostate cancer progression. Prostate.

[30] Ghadersohi A, Sood AK (2001). Prostate epithelium-derived Ets transcription factor mRNA is overexpressed in human breast tumors and is a candidate breast tumor marker and a breast tumor antigen. Clin Cancer Res.

[31] Lai KC, Liu CJ, Chang KW, Lee TC (2013). Depleting IFIT2 mediates atypical PKC signaling to enhance the migration and metastatic activity of oral squamous cell carcinoma cells. Oncogene.

[32] Li W, Hou G, Zhou D, Lou X, Xu Y, Liu S, Zhao X (2016). The roles of AKR1C1 and AKR1C2 in ethyl-3,4-dihydroxybenzoateinduced esophageal squamous cell carcinoma cell death. Oncotarget.

[33] Li C, Tian ZN, Cai JP, Chen KX, Zhang B, Feng MY, Shi QT, Li R, Qin Y, Geng JS (2014). Panax ginseng polysaccharide induces apoptosis by targeting Twist/AKR1C2/NF-1 pathway in human gastric cancer. Carbohydr Polym.

[34] Yu XM, Jaskula-Sztul R, Georgen MR, Aburjania Z, Somnay YR, Leverson G, Sippel RS, Lloyd RV, Johnson BP, Chen H (2016). Notch1 signaling regulates the aggressiveness of differentiated thyroid cancer and inhibits SERPINE1 expression. Clin Cancer Res.

[35] Zhu X, Guo X, Wu S, Wei L (2016). ANGPTL4 correlates with NSCLC progression and regulates epithelial-mesenchymal transition via ERK pathway. Lung.

[36] Zhou SL, Hu ZQ, Zhou ZJ, Dai Z, Wang Z, Cao Y, Fan J, Huang XW, Zhou J (2016). miR-28-5p-IL-34-macrophage feedback loop modulates hepatocellular carcinoma metastasis. Hepatology.

[37] Zhou ZJ, Dai Z, Zhou SL, Fu XT, Zhao YM, Shi YH, Zhou J, Fan J (2013). Overexpression of HnRNP A1 promotes tumor invasion through regulating CD44v6 and indicates poor prognosis for hepatocellular carcinoma. Int J Cancer.

