# Supplementary material for: Prostate-derived ETS factor improves prognosis and represses proliferation and invasion in hepatocellular carcinoma
Source: Oncotarget. 2017 Jan 31;8(32):52488–500. doi: 10.18632/oncotarget.14924 (PMC5581045; doi:10.18632/oncotarget.14924)
Supplement: Supplementary file 1 [file oncotarget-08-52488-s001.pdf]

Prostate-derived ETS factor improves prognosis and represses proliferation and invasion in hepatocellular carcinoma

SUPPLEMENTARY MATERIALS

RNA extraction

Trizol reagent (1 mL) was added to each sample, vortexed for 10-15s, and incubated at room temperature for 10 min to effectively denature proteins. Chloroform (0.2 mL) was added and mixed for 15s to separate the aqueous and the organic phase. All samples were incubated for 10 min at room temperature for the complete dissociation of nucleoprotein complexes. All samples were centrifuged at 12,000 ×g for 10 min at 4°C. Supernatant was transferred to a new tube and isopropanol (0.5.mL) added and incubated in -20 °C for 30 min. All tubes were centrifuged at high speed 16,000 ×g, 4°C for 10 min. The pellets were dried and dissolved in 50 µL RNA-free water.

Quantity and quality assessment of the RNAs

The RNA concentration, quantity, and purity were assessed using optical density at A260/280 and A260/230 on a spectrophotometer (NanoDrop Technologies, USA).

Protein extraction

The tissues from HCC patients undergoing liver resection at our institute were cut into 1 cm thick slices, rinsed three times with PBS to remove blood and ground in liquid nitrogen. Total proteins were extracted from tissue samples by RIPA containing protease inhibitors incubated at 0°C for 15 min. Insoluble debris was removed by centrifugation at 12,000 ×g for 30 min at 4°C.The supernatant was transferred to new tubes. 5X loading buffer was added and all samples were boiled for 5 min.

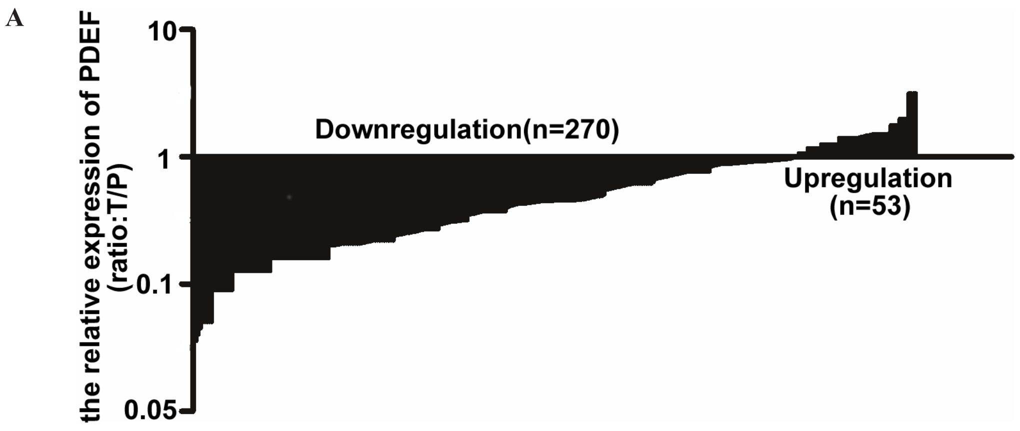

Supplementary Figure 1: A. **Relative expression of PDEF among tumor and peritumoral tissues in the TMA.**
